# Healthcare workers’ knowledge of indicators for a palliative care approach

**DOI:** 10.4102/phcfm.v16i1.4467

**Published:** 2024-07-31

**Authors:** Jennie Morgan, Ruth Amoore, Sadiya Z. Patel, Katya Evans, Rene Krause

**Affiliations:** 1 Department of Family, Community and Emergency Care, Division of Family Medicine, Faculty of Health Science, University of Cape Town, Cape Town, South Africa; 2 Metro District Health Services, Western Cape Government, Department of Health and Wellness, Cape Town, South Africa; 3 Faculty of Health Science, University of Cape Town, Cape Town, South Africa; 4 Department of Family, Community and Emergency Care, Division of Emergency Medicine, Faculty of Health Science, University of Cape Town, Cape Town, South Africa; 5 Department of Family, Community and Emergency Care, Division of Interdisciplinary Palliative Care and Medicine, Faculty of Health Science, University of Cape Town, Cape Town, South Africa

**Keywords:** palliative care, health care workers, South Africa, SPICT-SA, primary health care, district health

## Abstract

**Background:**

Palliative care is an essential element of universal healthcare, yet not all people who need palliative care are able to receive it. One of the barriers to ensuring access for people who require palliative care is the identification of those eligible.

**Aim:**

This study evaluated healthcare workers’ ability to identify patients who are eligible for palliative care based on their training or experience in palliative care.

**Setting:**

The setting for the study comprised the Heideveld Emergency Centre and Heideveld Community Day Centre in the Cape Metro, Cape Town, South Africa.

**Methods:**

This study made use of a cross-sectional survey of healthcare workers.

**Results:**

Of the 55 participants in this study, most were able to correctly identify patients with cancer and chronic kidney disease as needing palliative care, but less accurate with other organ failure categories, trauma indications, or functional assessment of the patient. Participants who reported previous awareness training reported improved knowledge on the indications for a palliative care approach compared to no prior training.

**Conclusion:**

Our cohort was too small to analyse the results statistically. From what was analysed, the ability of healthcare workers to identify a person in need of palliative care could be better; more work is needed on our awareness training and basic training courses to improve this vital step.

**Contribution:**

This research highlights the fact that existing training for palliative care needs to be more applicable to the setting and that training of staff with existing courses does make a difference in knowledge.

## Introduction

Palliative care is essential for people with life-limiting and life-threatening illnesses.^1^ Palliative care is a crucial element of universal healthcare and is recommended by the World Health Organization (WHO) as fundamental in the continuity of care. Temel and colleagues showed that early palliative care resulted in improved quality of life and longer survival.^2^

Healthcare workers play an important role in diagnosing and identifying patients with a life-limiting or threatening illness, as well as educating and counselling patients and their families about their conditions and initiating a palliative care approach. A significant responsibility that healthcare workers have is to ensure that patients requiring palliative care are identified promptly so that a palliative care approach can be initiated early. Healthcare workers need to know when to refer patients for palliative care to ensure patients receive the care timeously. This research will evaluate healthcare workers’ ability to identify patients needing palliative care in a primary care setting.

There are many tools or methods to identify patients who require palliative care. The Supportive and Palliative Care Indicators Tool – South Africa (SPICT-SA) (Online Appendix 1)^3^ was developed for South Africa using the Delphi process to adapt the SPICT.^4^

The tool is divided into two sections: general indicators and disease-specific indicators. The SPICT-SA highlights some of the general indicators in determining palliative care patients such as poor performance status of the patient, patients suffering from conditions with limited reversibility, dependency for care, severe weight loss within past 3–6 months, ineffective treatment and persistent symptoms, two or more unplanned hospital admissions in the past 6 months, and patient request to withdraw from treatment.^3^

These general indicators help healthcare workers to identify people with no diagnosis or a rarer diagnosis that is not listed on the SPICT-SA, but who would still benefit from receiving palliative care.

The disease-specific indicators are divided according to the major diseases causing mortality in South Africa: cancer, haematological diseases, infectious diseases, kidney diseases, lung diseases, heart/vascular diseases, liver diseases, neurological disease, dementia/frailty, trauma, and any other disease where the patient is deteriorating despite best available or appropriate treatment (See SPICT SA).^3^

The SPICT-SA has included tuberculosis (TB) and trauma-related deaths.

Despite indicators being available for a range of illnesses, many studies have shown that the majority of the people that receive palliative care are patients with cancer.^1,5,6^ Patients with HIV, end-stage organ failure, drug-resistant TB, and traumatic injuries often do not receive timeous palliative care.^5^ These two specific indicators are an important addition to the SPICT-SA, because in 2012, human immunodeficiency virus/acquired immune deficiency syndrome (HIV/AIDS) and TB accounted for 33.6% of all deaths in South Africa and trauma accounted for 9.6% of all deaths.^7^ People living with HIV, TB, and those experiencing severe trauma will benefit from palliative care.

There are still big gaps in education and training in palliative care in South Africa. There are several reasons for this. Firstly, training of undergraduate doctors and nurses in palliative care is not yet standardised across the country.^8^ Secondly, in South Africa, palliative care is still not recognised as specialised medical care leaving a gap in clinical leadership. Lastly, many healthcare workers qualified before training in palliative care was introduced into the undergraduate syllabus.^9^

These gaps are not found only in South Africa. The WHO was cognisant of these gaps in the 2014 World Health Assembly (WHA) resolution, stating that each country has the responsibility to ensure that all healthcare workers provide basic palliative care.^6^ This includes being able to identify which patients require palliative care, being able to assess the needs of patients and families, providing care that relieves distressing symptoms, and being able to identify when patients need more specialised care.

The European Association for Palliative Care (EAPC) gives guidance on the levels of palliative care training that are needed at each level of care. The levels of care they describe are as follows: palliative care approach, general palliative care, and specialist palliative care.^10^

A palliative care approach would be a way to integrate palliative care methods and procedures in settings not specialised in palliative care. This level of training should be made available to general practitioners and staff in general hospitals, as well as to nursing services and nursing home staff. It may be taught through undergraduate learning or through continuing professional development.

General palliative care should be provided by primary care professionals and specialists treating patients with life-threatening diseases who have good basic palliative care skills and knowledge. This level of training should be made available to professionals who are involved more frequently in palliative care, such as oncologists or geriatric specialists, but do not provide palliative care as the focus of their work. Depending on the discipline, this may be taught at an undergraduate or postgraduate level or through continuing professional development.

Specialist palliative care should be provided in services whose main activity is the provision of palliative care. These services generally care for patients with complex and difficult needs and therefore require a higher level of education, staff, and other resources. Specialist palliative care is provided by specialised services for patients with complex problems not adequately covered by other treatment options. This is usually taught at a postgraduate level and reinforced through continuing professional development.^10^

As basic palliative care training is a recent addition to some undergraduate training programmes, the basic knowledge of palliative care cannot be assumed to be present for the average healthcare worker in South Africa.

Healthcare providers need to be able to identify who would benefit from palliative care so that they can initiate palliative care timeously. This is especially important in the South African healthcare sector where the whole team is involved in the care of a patient who needs palliative care. Thus, other healthcare workers are encouraged to highlight to the busy doctors, where palliative care may be of benefit for a patient and family. Once a doctor has initiated palliative care, the rest of the multidisciplinary team can assist with the implementation. For example, offering nursing advice to the family or psychosocial support to the patient and family. Thus, all healthcare workers need to know when to implement palliative care and implement or refer for palliative care.

The need for palliative care is evident in the Heideveld Community Day Centre (CDC) in Cape Town, South Africa. The Heideveld CDC is an 8-h healthcare facility offering primary healthcare for the communities of Heideveld and Manenberg. The facility offers preventative, curative, rehabilitative, and palliative care services to the whole community. The Heideveld CDC works closely with the non-governmental organisations (NGOs) contracted to provide community health services to these disadvantaged and gang-controlled communities.^11^ This facility is also connected to Heideveld Emergency Centre (HEC), which is a 24-h emergency unit that offers district-level care to the district of Klipfontein, which includes Heideveld and Manenberg. Heideveld Emergency Centre also has a 30-bed ward attached to it, enabling them to provide a short duration of district-level inpatient care to the Klipfontein District. It is worth noting that the Klipfontein district has a high burden of trauma. In the 2013 Western Cape Mortality Profile, it lists interpersonal violence as the 5th leading cause of death; but across the province, it is the leading cause of premature deaths for men.^12^ In Klipfontein, interpersonal violence is the leading cause of premature death at 15.2%.^12,13^ It is worth noting that it has been projected by Sleeman et al., that injuries requiring palliative care are only going to increase by 2060, by an estimated 30%.^14^ These statistics underscore the need for palliative care in specific trauma-related scenarios.

In 2021, Heideveld CDC and HEC were identified as a palliative care training site, forming a partnership with the University of Cape Town (UCT) Division of Interdisciplinary Palliative Care and Medicine (IPCM) and Western Cape Department of Health and Wellness (WC DHW). The intention is to create a community site where quality palliative care is offered to the people who access care at these two facilities and then use the site to train all categories of Healthcare workers (HCWs), undergraduate, and postgraduate students in palliative care. University of Cape Town has been working closely with the two facilities and the community to begin to achieve this.

The Heideveld and Manenberg communities are of particular interest, as they are among the few suburbs in Cape Town that have been excluded from receiving palliative care from the Cape Town-based NGO hospices for many years, because of the gang activity and high incidence of trauma. These communities have few options regarding accessing healthcare; therefore, there is a great need to ensure that the healthcare that is available to them offers quality palliative care.^15^ The need is further amplified when we review the small proportion of the population receiving palliative care in comparison to the proportion of the population that requires palliative care. It is estimated that in South Africa, up to 50% of all deaths could benefit from a palliative care intervention.^16^ One cause for this disparity could be healthcare workers’ ability to correctly and timeously identify people in need of palliative care.

University of Cape Town, recognising the need to involve all parties from the outset,^16^ has collaborated with these two facilities and the community to integrate palliative care in training, service delivery, and research. As part of this integration, the research team aimed to describe the healthcare workers’ knowledge of indicators for initiating a palliative care approach, when a disease is diagnosed and when a specific disease is unknown; and to evaluate the effect of prior training and experience in palliative care on correctly identifying patients in need of a palliative care approach. This study highlighted the gaps in the participants’ current knowledge and the influence of training on identifying patients in need of palliative care correctly.

## Research methods and design

This is a cross-sectional survey, using the web-based Google forms, that was conducted with all categories of nursing and doctor staff at Heideveld CDC and HEC. In total, there are 98 staff members to sample, with Heideveld CDC having 29 nurses and 5 doctors and HEC having 48 nurses and 16 doctors.

Convenience sampling was used to recruit the participants, with all staff of above categories working during the duration of the study invited to participate. Attempts were made to invite all 98 staff members to participate; links to the online survey were sent via email and WhatsApp to all available staff, but it is unknown why some staff did not participate. No participant withdrew consent after the survey was completed.

The survey variables were based on the recommendations of the SPICT-SA tool consensus group^3^ (Survey tool available as Online Appendix 2) with a 3-point Likert rating scale. The survey comprised two parts. Part 1 comprised participant demographics, experience, and training, and Part 2 comprised mini-case vignettes, with questions 1–11 being disease-focused, and questions 12 to 15 were symptom-focused. Questions 1–15 were created by the researchers from prior experience of implementing the SPICT-SA tool. Three of the authors (J.M., K.E. and R.K.) were members of the Delphi study that developed the SPICT-SA tool (with R.K. being the first author).^3^ One of the team members has an MPhil in Palliative Care while the other two members both hold postgraduate diplomas in Palliative Medicine and teach undergraduate and postgraduate students in Palliative Medicine. They have more than 20 years of cumulative knowledge of initiating palliative care in the South African setting between them. These three authors were responsible for writing the 15 mini-case vignettes and agreeing on the correct answers.

The survey was piloted on three medical interns not included in the study population. Face validity was ensured by sharing the tool with experts in the field. Content validity was ensured by aligning it with common problems seen in both facilities and the SPICT-SA tool.

Data analysis was performed using STATA. Simple descriptive analysis was performed, with categorical variables being reported as proportions, as the sample size was too small to conduct more analyses that would explore statistical associations between variables.

### Ethical considerations

Approval from the University of Cape Town Human Research Ethics Committee was obtained (UCT HREC Reference 493/2022). As this was an undergraduate short study module, only local facility approval was obtained. Written consent was obtained during the completion of the online survey.

As this formed part of an undergraduate research study, the medical students assisted with the recruitment and data capturing of the online survey. This reduced the bias as they were not known to the participants prior to the study and thus there was no coercion to participate in the study. The students were not blinded to the study, but were not aware of the correct answers to the mini-case vignettes at the time of data collection.

## Results

This study took place in July 2022. In total, there were 55 healthcare workers who completed the online survey. Electronic links were sent to all 98 healthcare workers at the study site. Of the 43 other healthcare workers who did not complete the survey, the researchers can only account for three staff members. Two were members of the research team and excluded themselves from the survey, one was off on long-term sick leave. There were 13 (23%) men and 42 (76%) women. The age groups are shown in Table 1.

**TABLE 1:** Demographics of the participants and details of how the participants were grouped according to professional groups, palliative care training, and experience (*N* = 55).

Variable	Category	*n*	%
Age ranges (years)	20–29	16	29
	30–39	18	32
	40–49	10	18
	50–59	10	18
	> 60	1	1
**Professional groups** †			
**Type of health care worker and rank**			
Junior nurses		15	27
	Student nurse	1	-
	Enrolled nursing assistants	6	-
	Enrolled nurses	8	-
Senior nurses		22	40
	Professional nurses	18	-
	Clinical nurse practitioners	4	-
Junior doctors		4	7
	Student intern	1	-
	Interns	2	-
	Community service doctors	1	-
Senior doctors		14	25
	Medical officers	11	-
	Registrar – Family Medicine	1	-
	Consultant	2	-
**Level of palliative care training per professional group** ‡			
Palliative care training	Junior nurses	12	80
	Senior nurses	16	73
	Junior doctors	2	50
	Senior doctors	6	43
	**Total**	**36**	**65**
Palliative care awareness	Junior nurses	1	7
	Senior nurses	4	18
	Junior doctors	0	0
	Senior doctors	3	21
	**Total**	**8**	**15**
Palliative care approach training	Junior nurses	2	13
	Senior nurses	2	9
	Junior doctors	2	50
	Senior doctors	5	36
	**Total**	**11**	**20**
**Level of palliative care experience per professional group** ‡			
Palliative care experience	Junior nurses	10	67
	Senior nurses	13	59
	Junior doctors	3	75
	Senior doctors	10	71
	**Total**	**36**	**65**
Possible palliative care experience	Junior nurses	3	20
	Senior nurses	8	36
	Junior doctors	1	25
	Senior doctors	4	29
	**Total**	**16**	**29**
Palliative care experience	Junior nurses	2	13
	Senior nurses	1	5
	Junior doctors	0	0
	Senior doctors	0	0
	**Total**	**3**	**5**

† , Total cohort;

‡ , Professional group.

Due to the low number of participants, statistical analyses that explored associations or correlations were not possible; thus only numbers and percentages are presented in Table 1.

Of the 55 HCWs surveyed, 37 were nurses and 18 were doctors. These staff were categorised into four primary groups for purposes of data interpretation. Table 1 shows the composition of these groups. Clinical nurse practitioners are registered nurses who have advanced training in primary healthcare (PHC) and consult patients in PHC, within a restricted scope. Student interns are final year medical students. Interns are medical graduates in the first 2 years after completion of their medical degree. Community service doctors are medical graduates in the third year of work after completion of their undergraduate degree.

Table 1 also shows the level of training and the palliative care experience for each of the groups. The classification of palliative care approach, general palliative care, and specialist palliative care training is in line with the EAPC classification of palliative care training for healthcare workers:^10^

- Palliative care awareness training was classified as informal training and PACK (in-service) training.
- Palliative care approach training was classified as undergraduate, home-based care, and 40-h Introduction to Palliative Care training.
- General palliative care training was classified as the postgraduate diploma in palliative care and the certificate course in palliative nursing.
- Specialist palliative care training was classified as holding an MPhil in palliative care.

Palliative care experience was asked, and from all the answers, the exact extent of the palliative care experience was not clear; thus, we divided the experience into no palliative care experience, possible palliative care experience, and palliative care experience.

Table 2 portrays the responses of the participants to each case vignette by professional group. The questions that scored the highest marks were as follows, in descending order (top 5) – Q6, Q1, Q5, Q7, and Q8. The questions Q6 and Q7 are both around cancer patients. Questions Q1, Q5, and Q8 are all patients for palliative care with chronic kidney disease (CKD), congestive cardiac failure (CCF), and dementia respectively.

**TABLE 2:** Correct responses to case vignettes by professional group.

Number	Variable	Junior nurses		Senior nurses		Junior doctor		Senior doctor		Total correct	
		*n*	%	*n*	%	*n*	%	*n*	%	*n*	%
1	Chronic Kidney Disease (CKD) for Palliative Care (PC)	11.0	73	14.0	64	4	100	11	79	40.0	73
2	Acute Kidney Injury (AKI) – not for PC	2.0	13	12.0	55	4	100	8	57	26.0	47
3	Congestive cardiac failure (CCF) – for PC	7.0	47	9.0	41	3	75	13	93	32.0	58
4	Peripheral Vascular Disease (PVD) – not for PC	6.0	40	6.0	27	4	100	7	50	23.0	42
5	Liver failure – for PC	9.0	60	10.0	45	3	75	13	93	35.0	64
6	Cancer – for PC	9.0	60	16.0	73	4	100	14	100	43.0	78
7	Cancer – not for PC	5.0	33	16.0	73	4	100	9	64	34.0	62
8	Dementia – for PC	10.0	67	10.0	45	3	75	11	79	34.0	62
9	Geriatric – not for PC	2.0	13	6.0	27	3	75	2	14	13.0	24
10	Dementia – for PC	8.0	53	10.0	45	2	50	12	86	32.0	57
11	Trauma – for PC	8.0	53	10.0	45	2	50	10	71	30.0	55
12	Functional assessment (Fx) – for PC	2.0	13	1.0	5	0	-	0	-	3.0	5
13	Fx – not enough info	2.0	13	3.0	14	0	-	1	7	6.0	11
14	Fx – not enough info	0.0	-	1.0	5	0	-	0	-	1.0	2
15	Fx – for PC	0.0	0	0.0	0	0	0	1	7	1.0	2
-	Average score out of 15 (%)	5.4	36	5.6	37	9	60	8	53	6.4	43

The area that the cohort did the worst in was the functional assessment. Q12–15 all scored 11% or less for the whole cohort. Table 3 portrays the responses of the participants to the case vignettes by palliative care training.

**TABLE 3:** Correct responses to case vignettes by palliative care training.

Number	Variable	No palliative care training (*N* = 36)		Palliative care awareness (*N* = 8)		Basic palliative care training (*N* = 11)		Total correct (*N* = 55)	
		*N*	%	*n*	%	*n*	%	*n*	%
1	CKD for PC	25.0	63	6.0	75	9.0	81	40.0	73
2	AKI – not for PC	18.0	69	3.0	38	5.0	45	26.0	47
3	CCF – for PC	17.0	47	5.0	63	10.0	91	32.0	58
4	PVD – not for PC	14.0	39	3.0	38	6.0	55	23.0	42
5	Liver failure – for PC	22.0	61	5.0	63	8.0	73	35.0	64
6	Cancer – for PC	25.0	69	7.0	88	11.0	100	43.0	78
7	Cancer – not for PC	19.0	53	7.0	88	8.0	73	34.0	62
8	Dementia – for PC	16.0	44	8.0	100	10.0	91	34.0	62
9	Geriatric – not for PC	7.0	20	4.0	50	2.0	18	13.0	24
10	Dementia – for PC	15.0	42	7.0	88	10.0	91	32.0	58
11	Trauma – for PC	18.0	50	5.0	63	7.0	63	30.0	55
12	Fx – for PC	2.0	6	1.0	13	0.0	0	3.0	5
13	Fx – not enough info	4.0	11	1.0	13	1.0	9	6.0	11
14	Fx – not enough info	0.0	0	0.0	0	1.0	9	1.0	2
15	Fx – for PC	0.0	0	0.0	0	1.0	9	1.0	2
-	Average score out of 15 (%)	5.6	37	8.5	57	7.6	51	6.4	43

CKD, Chronic Kidney Disease; AKI, Acute Kidney Injury; CCF, Congestive Cardiac Failure; PVD, Peripheral Vascular Disease; PC, Palliative Care; FX, Functional assessment.

## Discussion

This research has affirmed many of the misconceptions regarding who requires a palliative care approach to be implemented. The results show that healthcare workers on the whole are good at identifying people with cancer and CKD as needing palliative care, yet very poor at identifying palliative care needs based on a functional assessment or those people in need of palliative care for other organ failure or trauma reasons. This study has also shown that training makes a difference in knowledge of when to initiate a palliative care approach, be it awareness training or basic training. While reported specific experience in palliative care does not seem to account for the differences, neither does general experience show a difference; for example, junior doctors scored on average higher than senior doctors. The study has addressed the aim of investigating healthcare workers’ knowledge of when to initiate a palliative care approach; but because of the small sample size, conclusions are limited. Inferences regarding training are possible from these results, but conclusions regarding experience are limited.

The main study findings were that participants could identify people with cancer and CKD as needing palliative care, but had trouble with other organ failure, trauma, and functional assessments which would identify people as needing palliative care. This is of concern in Heideveld where the need for palliative care for a broad range of conditions is vital. This finding is particularly relevant in low-income and middle-income countries where people dying from HIV, organ failure, TB, and traumatic injuries do not receive timeous palliative care.^5^ Our findings infer that health care workers do not know when to initiate palliative care for a wide range of common conditions, resulting in people not accessing palliative care. Specifically, the findings show that healthcare workers are not aware of the general indicators for palliative care that may apply to someone who does not have a specific disease diagnosed but is nearing the end of their life and should receive palliative care. More work is needed to upskill healthcare workers.

This study identified the gap in healthcare worker knowledge when reviewing the ability to identify trauma patients. This is especially important in the Heideveld, Manenberg, and Klipfontein districts that experience a high burden of trauma.^12^ It is recommended that trauma patients with severe burns and significant brain injuries should include a palliative approach in their care.^3^ This gap needs to be addressed in all forms of training to improve healthcare workers’ knowledge.

When comparing palliative care training, the data show that palliative care awareness does make a difference regarding cancer and dementia patient identification, compared to no training. Basic palliative care training makes additional difference in CKD and CCF patients, but no difference in other organ failure, trauma cases, or functional assessments. This is of concern in a country with a high burden of disease related to HIV and TB, organ failure, and traumatic death.^7^ Standardising the palliative care education across South Africa is still in process.^9^ According to these results, the current palliative care awareness and basic training is not assisting healthcare workers to identify patients with other organ failure, trauma, or functional assessments; more focus is needed on strengthening teaching in these areas.

The last section of the aims and objectives was to evaluate if experience in palliative care influenced healthcare workers’ knowledge of when to initiate a palliative care approach. With no statistical analysis, it is not possible to state if there was or was not a difference between the groups.

When looking at the overall results of all participants for the 15 questions, doctors scored, on average, more than the nurses (Senior doctors 8/15, Junior doctors 9/15, Senior nurses 5.6/15, and Junior nurses 5.4/15). A guiding principle in palliative care is that a team is needed to provide palliative care, not an individual healthcare worker.^17^ So, it is recommended that all members of the team should know when to implement palliative care and their opinion should be listened to.

At the site, there are three doctors who are trained in intermediate palliative care; however, they were either involved in this study as authors or not available to be recruited in this time frame; thus more trained healthcare workers were not included in these results and this may alter the results if they were to have been included. So, while this is not a true reflection of the palliative care capacity at Heideveld, it is a true reflection of the non-palliative care trained staff.

### Limitations

Due to the low cohort number, no statistically significant analysis could be performed on that data; thus only trends were commented on for the discussion. Of the possible 98 staff, only 55 were recruited for the study. There was a time limitation for this study and a larger cohort could be obtained if this study were to be repeated. It is true that the small sample size impairs representativity of the study population and reliability of the findings. This is an innate limitation of the chosen methodology: cross-sectional descriptive survey.

Recommendations would be:

- To recruit a large cohort.
- Our palliative care awareness training needs to touch on all indications for implementing palliative care including other organ failure, trauma, and functional assessments.
- This research does show that more staff members need to be trained in basic palliative care.
- Possible qualitative research to explore why our staff cannot identify other groups of people in need of palliative care, what are the barriers to this.
- This survey can be used as a baseline assessment of HCWs’ knowledge, training, and experience when starting a new palliative care service.

## Conclusion

The results of the baseline staff knowledge assessment demonstrated poor knowledge of the indications for a palliative care approach. Staff members are good at identifying cancer and dementia indicators for palliative care, but are less able to identify the need for palliative care for trauma victims. More training and mentorship is recommended to make Heideveld CDC and HEC a site that allows staff good palliative care experience, with a particular focus on nursing staff inclusion in the discussion or decision to implement a palliative care approach.
